# Origin of the Temperature Dependence of Gate-Induced Drain Leakage-Assisted Erase in Three-Dimensional nand Flash Memories

**DOI:** 10.3390/mi15121516

**Published:** 2024-12-20

**Authors:** David G. Refaldi, Gerardo Malavena, Luca Chiavarone, Alessandro S. Spinelli, Christian Monzio Compagnoni

**Affiliations:** 1 Dipartimento di Elettronica, Informazione e Bioingegneria, Politecnico di Milano, 20133 Milan, Italy; gerardo.malavena@polimi.it (G.M.); alessandro.spinelli@polimi.it (A.S.S.); christian.monzio@polimi.it (C.M.C.); 2 Process Research and Development Department, Micron Technology Inc., 20871 Vimercate, Italy; lchiavar@micron.com

**Keywords:** nand Flash memory, 3D array, gate-induced drain leakage, semiconductor device modeling

## Abstract

Through detailed experimental and modeling activities, this paper investigates the origin of the temperature dependence of the Erase operation in 3D nand flash arrays. First of all, experimental data collected down to the cryogenic regime on both charge-trap and floating-gate arrays are provided to demonstrate that the reduction in temperature makes cells harder to Erase irrespective of the nature of their storage layer. This evidence is then attributed to the weakening, with the decrease in temperature, of the gate-induced drain leakage (GIDL) current exploited to set the electrostatic potential of the body of the nand strings during Erase. Modeling results for the GIDL-assisted Erase operation, finally, allow not only to support this conclusion but also to directly correlate the change with temperature of the electrostatic potential of the string body with the change with temperature of the erased threshold-voltage of the memory cells.

## 1. Introduction

Three-dimensional nand Flash technology represents the leading solution for nonvolatile data storage [1,2,3,4,5] thanks to its very low cost per bit, which is the direct outcome of its ability to reach an extremely high storage density with a cost-effective process [6,7,8,9]. One of the most relevant innovations behind that capability is the integration of the 3D memory array over an $n^{+}$-doped polysilicon source plate on top of the CMOS peripheral circuitry [8,10,11,12]. In spite of the benefits of this integration scheme, the lack of any connection of the nand strings to a *p*-doped bulk contact makes the exploitation of gate-induced drain leakage (GIDL) at the string edges the only viable option to provide the string body with the holes needed during an Erase operation. GIDL-assisted Erase has been the object of detailed investigations in the past, which successfully highlighted its time dynamics through both TCAD and compact models [10,13,14]. However, some aspects of this operation, such as its temperature (*T*) dependence, still require better explorations, especially for *T* values ranging from room temperature (RT) down to the deep-cryogenic regime (T<50 K). Indeed, cryogenic operation of 3D nand Flash memories has been recently reported as a promising candidate for future high-performance applications [15,16,17,18,19]. Nevertheless, to date, it is still not entirely clear what impact temperature has on the processes underlying the performance and reliability of memories when they are operated at cryogenic temperatures. The GIDL-assisted Erase of 3D nand Flash memories is an operation that leverages on a large number of physical processes. As each of these features a specific *T* dependence, the identification of the main process controlling the *T* activation of the Erase is a mandatory prerequisite to extend the reliable operation of devices out of nominal *T* conditions. Although it is known that the GIDL current features a positive *T* activation [10,20,21,22,23], such dependence has not been explored down to cryogenic temperatures. Moreover, it remains unclear if the GIDL current is the dominant *T*-activated phenomenon during Erase. Indeed, a recent work put forward the hypothesis that it is the emission of electrons from the charge-trap layer that determines the main thermal activation of Erase [16].

In this paper, we experimentally investigate, for the first time, GIDL-assisted Erase in 3D nand Flash arrays over an extremely wide *T* interval from 300 K down to the cryogenic regime and on both charge-trap (CT)- and floating-gate (FG)-based memory cells. We provide clear experimental evidence showing that Erase becomes more demanding in terms of the voltage needed to reach a target cell threshold voltage ($V_{T}$) when *T* is reduced. The observation that the nature of the cell storage layer does not impact the *T* dependence of the Erase operation allows the identification of the main factor responsible for the observed slow-down in the weakening of the GIDL current when *T* decreases. Detailed modeling results allow to confirm this view and to correlate the *T*-induced change of the electrostatic potential of the string body during an Erase pulse with the *T*-induced change of the erased $V_{T}$ of the memory cells.

## 2. Experimental Section

**Figure 1:**
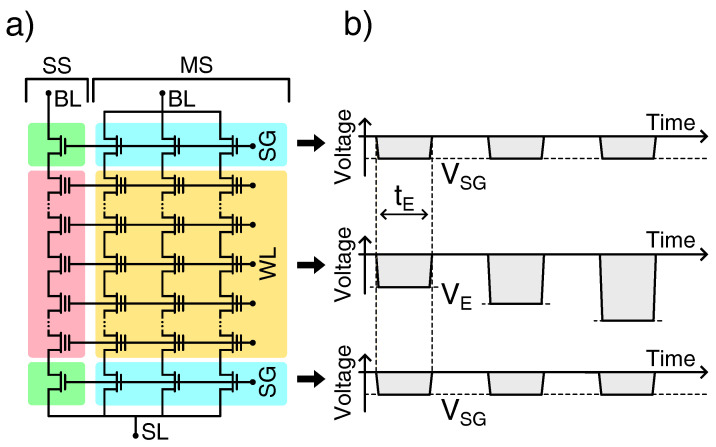
(**a**) Schematic description of the test elements investigated in this work (only some of the WLs are depicted in the scheme). (**b**) Voltage waveforms applied to the array WL and SG to achieve ISPE, with grounded SL and BL ($t_{E}$ is the duration of the Erase pulses).

The samples investigated in this work are 3D nand Flash array test elements based on charge-trap (CT) and floating-gate (FG) storage with, respectively, $N_{WL}=10$ and ≫10 vertically stacked wordline (WL) layers. To offer the opportunity to monitor either a single string (SS) or many strings (MS) in parallel, the contacts of some of the bitlines (BL) are independently available for biasing, while those of some others (a few thousands) are short-circuited, as schematically shown in Figure 1a.

To explore the Erase operation over a wide *T* interval ranging from 300 K to about 15 K, the investigated samples were encapsulated in a package and mounted in a cryostat, as described in [24]. An Incremental Step Pulse Erasing (ISPE) scheme was used, consisting of negative voltage pulses of increasing amplitude $V_{E}$ applied to the array WLs and negative voltage pulses of constant amplitude $V_{SG}$ applied to the select-gate (SG) lines, with grounded BLs and sourceline (SL) (see Figure 1b). Note that the application of negative pulses to the array WL and SG is perfectly equivalent to the application of positive pulses to the BL, SL, and SG; although the latter solution represents the standard way to perform the Erase operation in 3D nand arrays, the former allows to simplify the experimental setup needed to carry out all operations on the investigated test elements. All cells were Erased well below their neutral state, i.e., the state in which no charge is present inside the storage node.

**Figure 2:**
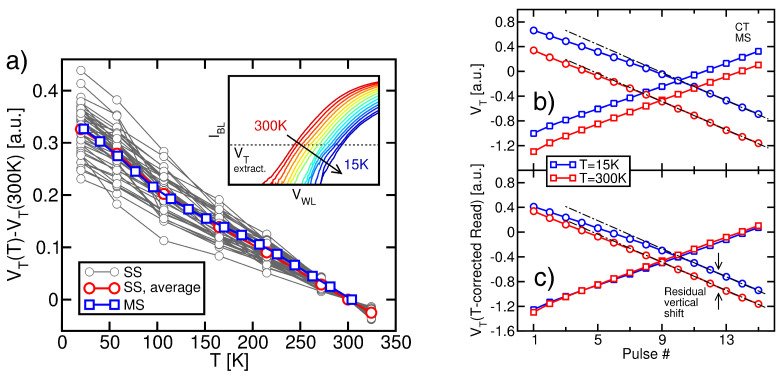
(**a**) *T*-induced change of cell $V_{T}$, with respect to 300 K. Data are reported for 50 SS and 1 MS measurements on CT-based cells. The average of the SS measurements is also highlighted. The inset shows a representative example for the $I_{BL}-V_{WL}$ trans-characteristic of the MS structure, at different *T* values (a log scale is used for $I_{BL}$). (**b**) $V_{T}$ evolution with the ISPE pulse number at T=300 K and 15 K, as obtained from MS measurements on CT-based samples (circles). ISPP results are also shown (squares). (**c**) Same as in (**b**) but with the *T* dependence of the Read operation subtracted from the $V_{T}$ values at T=15 K. Dashed black lines are guides to the eye. All the voltages reported in this work were normalized to the same arbitrary constant.

After each ISPE pulse, the BL current vs. WL voltage ($I_{BL}-V_{WL}$) trans-characteristic of single cells was measured, with all the other WLs under the pass condition, positively biased SG and BL, and grounded SL. When $I_{BL}$ is read through an SS contact, the curve obtained via this measurement allows the extraction of the $V_{T}$ of a single cell in the sub-block under test. When $I_{BL}$ is read through an MS contact, instead, the curve provides an equivalent $V_{T}$ of many cells in parallel. In both cases, $V_{T}$ is defined as the $V_{WL}$ value leading to a fixed $I_{BL}$ value well below the minimum saturation level of the string current at T=15 K. An example of the $I_{BL}-V_{WL}$ trans-characteristic resulting from an MS measurement at different *T* values is reported in the inset in Figure 2a for a CT-based sample, highlighting the $I_{BL}$ threshold value. The rightward shift in the trans-characteristic with the reduction in *T* results in the typical increase in $V_{T}$ observed in all MOS devices down to the cryogenic regime [25,26,27,28,29,30,31]. The change in $V_{T}$ with respect to the 300 K value is reported as a function of *T* in Figure 2a along with data from 50 SS measurements. Clearly, the trend obtained from the MS measurement is very similar to the average trend obtained from the latter, confirming that MS measurements allow us to achieve results representative of single cell behavior and to avoid the statistical variability and noise typical of SS results.

Figure 2b (circles) shows the $V_{T}$ evolution with the ISPE pulse number, as obtained from an MS measurement on a CT-based sample at T=300 K and 15 K (both Read and Erase operations were carried out at the same *T*). Although $V_{T}$ features a linearly decreasing trend with a *T*-independent slope, the curve at 15 K is significantly above the one at 300 K. This can be only partially attributed to the *T* dependence of the $V_{T}$ value resulting from a Read operation (see Figure 2a). Figure 2c shows, in fact, that the 15 K curve remains clearly above the 300 K one even when the *T* dependence of the Read operation is subtracted from its $V_{T}$ values (this correction was performed by vertically shifting the transients measured at T=15 K by a quantity equal to the *T*-induced shift in the neutral $V_{T}$). This demonstrates that cells become harder to Erase with the reduction in *T*, in agreement with what was reported in [16,17] where data were gathered down to 77 K. Cell programming, instead, is not made significantly harder by the reduction of *T*: Figure 2b (squares) shows, in fact, that when an Incremental Step Pulse Programming (ISPP) scheme is adopted [32,33], the $V_{T}$ transient at T=15 K is still above that at 300 K but overlaps with it when the *T* dependence of $V_{T}$ is accounted for. This indicates that the transfer of negative charge from the channel to the storage layer of the memory cells during Program remains almost the same irrespective of *T*, meaning that the *T* dependence of Fowler–Nordheim tunneling is very weak [16,17].

**Figure 3:**
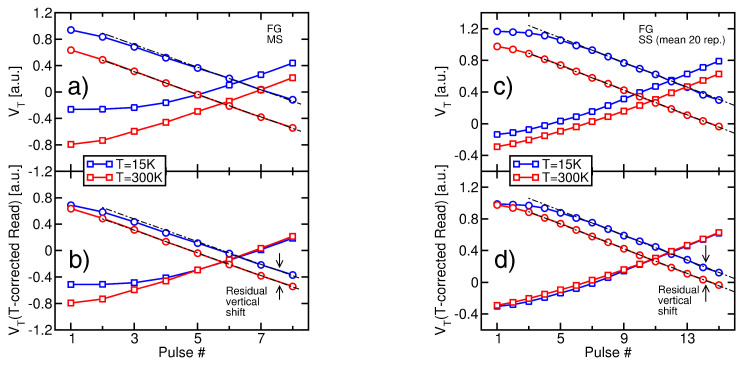
(**a**,**b**) Same as in Figure 2b,c, but for FG-based cells. (**c**,**d**) Same as in Figure 2b,c but for an average of 20 SS measurements on FG-based cells.

Figure 3a,b proves that the same evidence gathered on CT-based samples also appears for FG-based devices. Note that the discrepancy present in the ISPP curves in Figure 3b for the first three pulses comes from the different initial placements of the cells at *T* = 300 K and 15 K. However, when constant-current operation is reached, such discrepancy is removed as the result of the convergent nature of the ISPP scheme [33]. The fact that the worsening of the Erase performance appears on FG-based samples is particularly important because it shows that the *T* dependence of the Erase operation in 3D nand Flash arrays does not trace back to something specifically related to the storage layer. For instance, it cannot be ascribed to the limited number of microscopic defects storing charge in the CT layer and neither to the *T* dependence of their time constant for carrier capture/emission [34,35,36,37]. Nor it is to be traced back to the fact that in the case of CT samples, cell Erase is the result of both electron emission from the storage layer and hole injection in the opposite direction [16,17,38]. In this regard, moreover, it is worth noting that the quantum–mechanical tunneling of holes from the channel to the storage layer of the memory cells is surely involved in the Erase operation of CT-based samples, being the only physical mechanism able to move $V_{T}$ below its neutral value. However, the *T* dependence of this mechanism is expected to be very weak, in analogy to the case of electron tunneling from the channel to the storage layer during Program. Therefore, neither the structure of the storage layer nor the physical processes involved in the charge transfer can be considered as the origin of the *T* dependence of the Erase operation. Also, because such *T* dependence is similarly observed for the largely different string lengths of the investigated CT and FG samples, that origin can neither be related to nonuniformities in the electrostatic potential along the string body during the Erase pulses, which may arise from electron/hole capture and emission at the polysilicon grain boundaries [39,40,41].

Finally, Figure 3c,d shows that the same trends obtained through the MS measurements were obtained through the average of SS measurements as well, confirming that the parallel Read of many memory cells does not introduce any artifact into the observed phenomenology.

## 3. Modeling

### 3.1. Physical Picture

A common origin for the *T* dependence of the Erase operation in CT- and FG-based arrays can be traced back to the change with *T* of the GIDL current setting the string electrostatic potential during the Erase pulses. In particular, a weakening of the GIDL current at the string edges with the reduction of *T* may easily explain the lower effectiveness of the Erase operation at T=15 K with respect to 300 K appearing from all the results presented in Section 2. That weakening, in fact, makes the electrostatic potential in the string body ($V_{B}$) during the Erase pulses more negative at the former than at the latter *T*. For the same negative $V_{E}$ applied at the cell WL, this results in a voltage drop over the cell gate stack that is lower at 15 K than at 300 K, making the Erase operation less effective.

**Figure 4:**
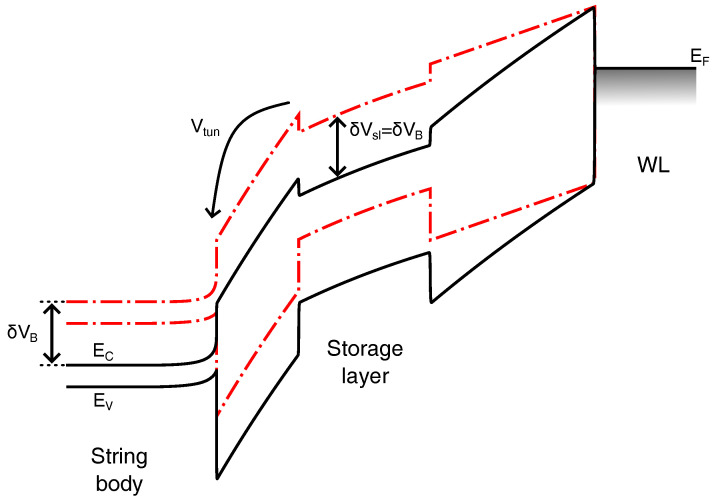
Schematic band diagram along the gate stack of a CT-based memory cell during an ISPE pulse at T=300 K (solid black curves) and 15 K (dashed red curves). $E_{C}$ and $E_{V}$ are, respectively, the conduction band and valence band edge of the materials. $E_{F}$ is the Fermi level in the metal WL. For the sake of simplicity, the tunnel–dielectric stack is considered as a single-layer dielectric.

To better visualize the impact of $V_{B}$ on the Erase operation, Figure 4 schematically shows the band diagram along the gate stack of a memory cell during an Erase pulse at T=15 K and 300 K for the same applied $V_{E}$. Direct reference is made to the ISPE scheme adopted in Section 2 and, in particular, to the part of this scheme during which $V_{T}$ displays a linear decrease with the Erase pulse number. When this condition is reached, each ISPE pulse gives rise to the same change in charge in the cell storage layer. This means that the average current flowing through the cell tunnel dielectric and the average voltage drop over this dielectric ($V_{tun}$) remain constant from one Erase pulse to the next. Moreover, under the assumption that the current vs. voltage characteristic of the tunnel dielectric during Erase does not change with *T* (in the case of dominant tunneling processes, the *T* dependence of such characteristic should be very weak, as discussed in the previous Section), the average value of $V_{tun}$ must be the same at T=15 K and 300 K. The change in $V_{B}$ ($\delta V_{B}$) between the two *T* arising from a different GIDL current at the string edges, then, has to turn into an equal change in the electrostatic potential in the cell storage layer ($\delta V_{sl}=\delta V_{B}$). This results in less positive charge in the cell storage layer at T=15 K, meaning that the Erase operation is less effective at low temperatures.

To determine the change in the erased cell $V_{T}$ arising from the *T*-induced $\delta V_{B}$, the following approximated expression for $V_{tun}$ during an Erase pulse can be used (the expression generalizes what was reported in [42]):

(1) $$V_{tun}=-\alpha_{G}\left(V_{E}-V_{B}-\Delta V_{T}+\beta \right),$$

where $\Delta V_{T}$ is the cell $V_{T}$ shift from the neutral value, $\alpha_{G}$ is the capacitive coupling ratio between the WL and the storage layer, and β is a constant accounting for the built-in voltage drop in the device. From (1), it is evident that a *T*-induced $\delta V_{B}$ must be compensated by an equal and opposite change in $\Delta V_{T}$ to maintain the same $V_{tun}$ during the ISPE operation. A negative $\delta V_{B}$ during the Erase pulses between T=15 and 300 K gives rise, then, to a higher erased $V_{T}$ at the former *T*. This not only explains the experimental evidence in Figure 2c and Figure 3b,d but also allows us to state that the magnitude of the vertical shift in the Erase transients, once corrected by the *T* dependence of the Read operation, quantitatively corresponds to the *T*-induced $|\delta V_{B}|$ during the ISPE pulses at T=15 K and 300 K.

### 3.2. Simulation Results

**Figure 5:**
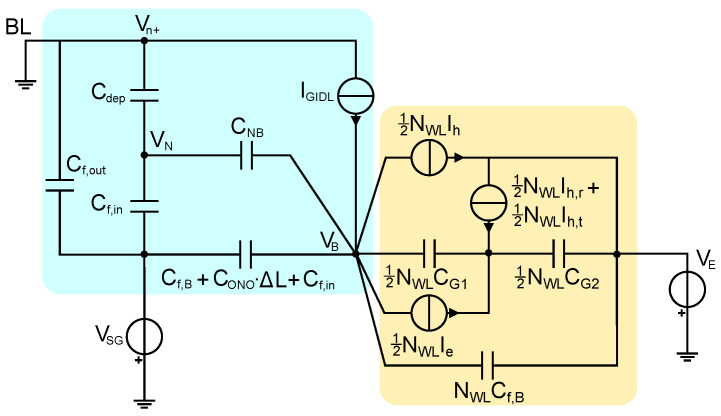
Compact model for GIDL-assisted Erase in CT-based 3D nand Flash strings, adapted from [13,14]. The light-blue region corresponds to the part of the model reproducing the $V_{B}$ dynamics in the presence of the GIDL current, while the yellow region corresponds to the part of the model reproducing the dynamics of charge transfer and storage in the gate stack of the cells. Note that only half of the string is considered in the model thanks to its symmetry.

To check the validity of the physical picture presented in Section 3.1 and confirm the conclusions drawn from (1) on the equality of the *T*-induced change in $\Delta V_{T}$ and $V_{B}$ during an ISPE operation, we relied on the compact model for GIDL-assisted Erase in CT-based 3D nand Flash strings presented in [13,14]. The model was originally developed to carefully reproduce the time dynamics of $V_{B}$, $I_{BL}$, and $V_{T}$ during a single Erase pulse and was here extended to reproduce the entire ISPE scheme. Figure 5 shows the lumped-parameter circuit at the heart of the model, which includes a module reproducing the $V_{B}$ dynamics in the presence of the GIDL current (light blue region) and a module reproducing the dynamics of charge transfer and storage in the gate stack of the cells (yellow region). In the circuit, $I_{GIDL}$ is the GIDL current providing the string with the holes needed to fix $V_{B}$ during the Erase pulse. $I_{h}$ represents the total hole current flowing from the channel to the WL of a memory cell. A fraction of this current results in holes trapped in the cell storage layer ($I_{h,t}$), and another fraction results in holes recombining with stored electrons ($I_{h,r}$) (all the remaining current corresponds to holes reaching the WL and not contributing to cell Erase [43]). $I_{e}$ is the total current arising from the release of electrons from the storage layer to the channel of the memory cell. Details about the functional form of the previously mentioned currents are reported in [14], along with the physical meaning of all the capacitive terms in the model. As a final remark, note that a negative WL voltage $V_{E}$ with grounded BL was assumed for Figure 5 to keep the same voltage convention as used in Section 2 and Section 3.1 (differently from [13,14], where a positive voltage was applied to the BL with grounded WL).

**Figure 6:**
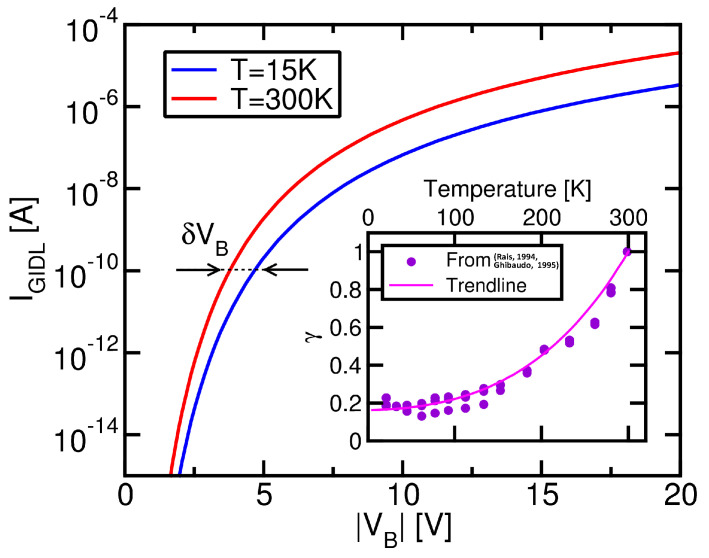
$I_{GIDL}$ vs. $V_{B}$ characteristics assumed in the compact model simulations at T=300 K and 15 K. The inset shows the factor γ determining the decrease in $I_{GIDL}$ with the reduction of *T* from 300 K, as obtained from experimental data collected on planar bulk MOSFETs [44,45].

Table 1 shows the parameters of the template nand string considered in this compact model, matching that used in [13,14]. Trapezoidal pulses were assumed in the simulations, with rise/fall time and plateau duration equal to 10 μs and 1 ms, respectively. $V_{E}$ was set to −10 V in the first pulse and then increased by $V_{s}=-0.5$ V per pulse. To address the dependence of the $V_{T}$ transient on *T*, the $I_{GIDL}$ characteristics were scaled by a factor γ moving from 300 K to a lower *T* value (no other parameter was changed in the simulations when changing *T*). This factor was obtained from results in the literature collected on planar bulk MOSFETs [44,45], reported in the inset in Figure 6. In this regard, it is worth noting that non-negligible differences exist between the GIDL phenomenology in planar bulk MOSFETs and thin-channel cylindrical nand strings: In the former devices, in fact, the GIDL current arises from vertical band-to-band tunneling within the drain junction of the transistor [46,47,48,49]. In the latter, instead, the band-to-band tunneling process occurs along the longitudinal direction at the $n^{+}$ edges of the string due to the weak band bending achievable in the radial direction [13,50,51]. Although some differences may in principle also exist in the *T* dependence of the GIDL current of the two types of devices, we relied on the available data on MOSFETs owing to the lack of available results on nand strings. A direct exploration of the GIDL current vs. voltage characteristics in nand strings, in fact, is precluded by the floating body and bipolar effects coming into play due to the $n^{+}$ contacts at both the string edges [50,52,53,54,55]. With this caveat, Figure 6 reports the $I_{GIDL}$ vs. $V_{B}$ characteristics assumed in our compact model simulations. Note that a quantitative match between the experimental data and model was outside of the scope of this work, which instead highlights the impact that the *T*-induced change in the GIDL current has on the resulting Erase transient. For this reason, we chose not to introduce any other *T* dependence in the compact model. Moreover, the simulation results remained valid regardless the specific *T* dependence of the GIDL current, which only determines the quantitative value of $\delta V_{B}$.

**Figure 7:**
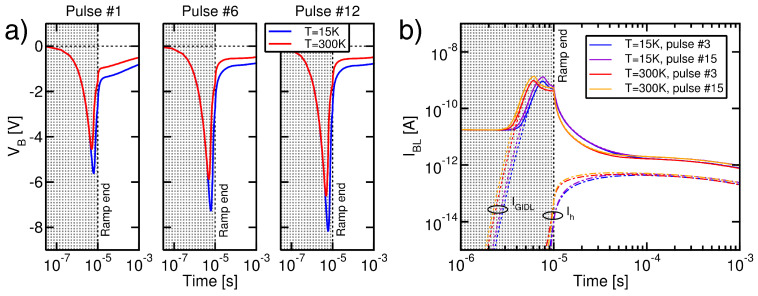
(**a**) Simulated $V_{B}$ transient during ISPE pulses at T=300 K and 15 K. The grey and white regions correspond, respectively, to the pulse front and plateau. (**b**) Simulated $I_{BL}$, $I_{GIDL}$, and $I_{h}$ during two Erase pulses of the ISPE scheme. The grey and white regions correspond, respectively, to the pulse front and plateau.

Figure 7a shows the simulated time evolution of $V_{B}$ during some of the pulses of the ISPE scheme at T=300 K and 15 K. The results reveal, first of all, that during each Erase pulse $V_{B}$ displays a rapid drop, followed by a relevant rise during the first front of the pulse and then a slow growth during the pulse plateau (see [13,14] for further details on this trend). The $V_{B}$ transient, additionally, displays just a very weak dependence on the pulse number. In agreement with the physical picture discussed in Section 3.1, moreover, $V_{B}$ is more negative at T=15 K than at 300 K. This is a direct consequence of the weakening of $I_{GIDL}$ at the former *T*, which introduces the only *T* dependence in the simulations. In this regard, Figure 7b shows that the simulated $I_{BL}$, which corresponds to $I_{GIDL}$ soon after the pulse plateau, spans the same values at both temperatures. This is, in the end, expected from the negative feedback setting $V_{B}$ as a result of $V_{E}$ and $I_{GIDL}$ [13,14] and shows that $\delta V_{B}$ during the Erase pulse nearly matches the horizontal shift in the curves in Figure 6 at the current levels involved in Figure 7b. As a final remark, note that Figure 7b also shows that the hole flow from the channel to the storage layer of the memory cells represents the dominant Erase mechanism in our simulations, making $I_{h}$ much higher than $I_{e}$.

**Figure 8:**
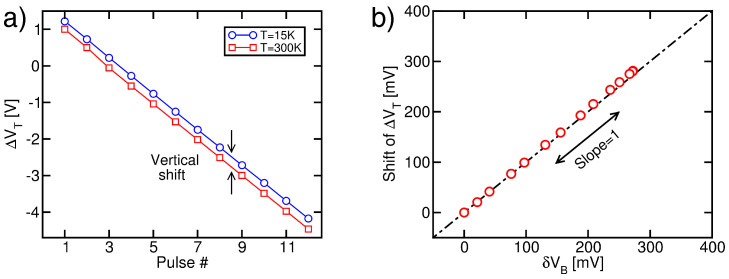
(**a**) Simulated $\Delta V_{T}$ transients during ISPE at T=15 K and 300 K. (**b**) Scatter plot of the vertical shift in $\Delta V_{T}$ transient with *T* and its corresponding $\delta V_{B}$ for different *T* values between 15 K and 300 K. The black dashed line is a guide to the eye, highlighting a one-to-one correlation.

The $\Delta V_{T}$ transients resulting from the compact model simulations are reported in Figure 8a. A linear trend with the pulse number clearly appears, in agreement with the fundamentals of the ISPE scheme. Additionally, in agreement with the experimental observations reported in Figure 2c and Figure 3b,d, the Erase operation is less effective at T=15 K than at 300 K. Once again, it is worth remarking that this result was obtained with our compact model through a weakening of the GIDL current only, with no thermal activation of the charge flows through the cell tunnel dielectric during the Erase pulses. Figure 8b demonstrates that the vertical shift in the $\Delta V_{T}$ transients at different *T* values well matches the corresponding values of $\delta V_{B}$ for all the simulations in the range of 15 K to 300 K ($\delta V_{B}$ was calculated by considering the $V_{B}$ value at the end of the plateau of the Erase pulses). This confirms the physical picture discussed in Section 3.1 with a rigorous modeling approach.

## 4. Conclusions

In this work, we investigated the origin of the *T* dependence of the Erase operation in 3D nand Flash arrays through experimental and modeling activities. We showed that such origin can be traced back to the *T* dependence of the GIDL current setting $V_{B}$ during the Erase pulses. In particular, a weakening of the GIDL current with the reduction in *T* allows to easily explain the lower effectiveness of the Erase operation observed when *T* decreases down to the cryogenic regime. Finally, the *T*-induced change in $V_{B}$ was directly correlated to the *T*-induced change in $V_{T}$ obtained from an ISPE scheme.

## Figures and Tables

**Table 1:** String parameters assumed in our compact model simulations. $N_{D}^{B}$ and $N_{D}^{BL}=N_{D}^{SL}$ are, respectively, the donor doping concentrations of the string channel and of the upper and lower $n^{+}$ regions. $L_{WL}$, $L_{SG}$, and $L_{S}$ are the WL, SG, and WL spacing lengths. $\phi_{m}$ is the WL work function; $r_{f}$, $t_{Si}$, and $t_{WL}$ are the filler oxide, channel, and WL thicknesses in the radial direction. $t_{O1}/t_{N}/t_{O2}$ are the thicknesses of the oxide/nitride/oxide stack used as gate dielectric.

$N_{WL}$	10	$L_{WL}=L_{SG}$	50 nm
$L_{S}$	50 nm	$r_{f}$	17.5 nm
$t_{Si}$	10 nm	$t_{O1}$/$t_{N}$/$t_{O2}$	4/4/4.5 nm
$t_{WL}$	40 nm	$N_{D}^{BL}=N_{D}^{SL}$	$5\cdot 10^{19}$ cm^−3^
$N_{D}^{B}$	$10^{15}$ cm^−3^	$\phi_{m}$	4.8 eV

## Data Availability

The original data presented in the study are not available due to confidentiality reasons.
